# The olfactory functional network in the Alzheimer’s disease continuum: a resting state fMRI study

**DOI:** 10.3389/fnagi.2025.1744413

**Published:** 2026-01-13

**Authors:** Daniela Ballotta, Claudia Casadio, Manuela Tondelli, Vanessa Zanelli, Francesco Ricci, Omar Carpentiero, Fausta Lui, Nicola Filippini, Annalisa Chiari, Maria Angela Molinari, Francesca Benuzzi

**Affiliations:** 1 Department of Biomedical, Metabolic and Neural Sciences, University of Modena and Reggio Emilia, Modena, Italy; 2 Neurology Unit, Azienda Ospedaliero-Universitaria (AOU) of Modena, Modena, Italy; 3 IRCCS San Camillo Hospital, Venice, Italy; 4 Azienda Ospedaliero-Universitaria (AOU) of Modena, Modena, Italy

**Keywords:** Alzheimer’s disease, functional connectivity, Mild Cognitive Impairment, olfactory dysfunction, resting-state fMRI

## Abstract

**Introduction:**

Olfactory dysfunction is common in the Alzheimer’s Disease continuum, and olfaction may be altered before clinical syndrome onset. The present study aimed at investigating the functional connectivity of the olfactory cortex and its correlation with olfaction performance in a group of patients with Mild Cognitive Impairment (MCI) who subsequently converted or not converted to Alzheimer’s Disease (AD) dementia.

**Methods:**

At baseline, 30 MCI patients were evaluated with the Sniffin’ Sticks (threshold, discrimination, and identification) to assess olfactory capacities, and they were followed up over time to identify converter and stable patients. Resting-state fMRI data acquired at baseline were analyzed to assess functional connectivity of left and right olfactory cortex. Beta values were extracted from the stable versus converter contrasts and correlated with olfactory scores.

**Results:**

Functional connectivity of the olfactory cortex was significantly increased with the posterior cingulate cortex, and significantly decreased with middle cingulate cortex, supplementary motor area, and left pre- and postcentral gyri, in converter compared to stable patients. Reduced negative functional connectivity between olfactory cortex and left angular gyrus emerged in converter patients, and a negative correlation was found between angular gyrus and discrimination scores.

**Discussion:**

Our findings indicate alterations of functional connectivity of the olfactory cortex in subjects with MCI at risk of conversion to AD dementia, even at the early stages of the disease. Additionally, the negative correlation between olfactory ability and the angular gyrus functional connectivity, a cerebral region known to be involved in multisensory integration processing, may be considered as a marker of disease progression.

## 1 Introduction

Age-related olfactory dysfunction is defined as a reduction in the capacity to detect, identify, or recall odors occurring during aging (Doty and Kamath, 2014). Interestingly, olfactory impairment is considered an early and common symptom in Alzheimer’s Disease (AD; Bathini et al., 2019; Son et al., 2021), and it has also been well documented in patients with Mild Cognitive Impairment (MCI; Dong et al., 2023; Roalf et al., 2017; Roberts et al., 2016; Vyhnalek et al., 2015). Indeed, behavioral studies demonstrated that olfactory impairment in MCI patients is linked to a more rapid cognitive decline (Dintica et al., 2019) and an elevated risk of progression to dementia (Daulatzai, 2015; Knight et al., 2023; Roberts et al., 2016).

Recent studies suggest that the olfactory deficit may reflect AD-related brain changes, spanning from cerebral atrophy (Chen et al., 2022; Zhan et al., 2025) and functional connectivity abnormalities (Xie et al., 2024; Yan et al., 2022) affecting smell related structures. In humans, the primary olfactory cortex (POC) encompasses the cortical targets of olfactory bulb projections—including the anterior olfactory nucleus, the olfactory tubercle, the frontal and temporal piriform cortices, and subregions of the amygdala and entorhinal cortex (Insausti et al., 2002; Milardi et al., 2017). In MCI and AD patients, structural neuroimaging studies have shown reduced gray matter volumes of the olfactory bulb, POC, and hippocampus (Murphy et al., 2003; Papadatos and Phillips, 2023; Thomann et al., 2009; Wu et al., 2019; Yi et al., 2022); this volumetric reduction is detectable as early as the MCI stage and it becomes more severe at the dementia stage (Jobin et al., 2021). Additionally, associations have been found between changes in brain volume of POC and odor identification ability (Vasavada et al., 2015). A reduction in gray matter volume of entorhinal and piriform cortices, hippocampus, and amygdala has also been reported in subject with Subjective Cognitive Decline (Chen et al., 2022), along with a correlation between entorhinal cortex atrophy and olfactory functions (Papadatos and Phillips, 2023). Indeed, the atrophy of olfactory-related brain regions seems to progress, accompanied by a gradual decline in olfactory function, along the continuum from healthy aging to SCD and MCI (Zhan et al., 2025).

Task-based fMRI studies in healthy subjects have revealed a complex cerebral network, known as the Olfactory Network (ON), involved in odor identification, valence and intensity processing (Torske et al., 2022). This network includes the POC and secondary olfactory areas, such as the hippocampus, insula, striatum, precuneus, and thalamus (Carlson et al., 2020; Georgiopoulos et al., 2019; Karunanayaka et al., 2014; Seubert et al., 2013). In healthy subjects, the ON has a strong functional interplay with the Default Mode Network (DMN; Raichle et al., 2001), with olfactory stimulation leading to transient DMN deactivation, likely reflecting cognitive and mnemonic demands of odor processing (Gottfried, 2010). Distinct connectivity profiles across POC subregions have been recently identified using resting-state fMRI (Zhou et al., 2019), reflecting the complexity of olfactory network organization. In MCI and AD dementia, structural abnormalities are often accompanied by functional changes in olfactory-related regions (Feng et al., 2021; Steffener et al., 2021; Wang et al., 2010; Zhu et al., 2023), with weaker activation of POC, hippocampus, and insula reported in AD patients compared to healthy controls during the administration of olfactory stimuli (Chen et al., 2022; Steffener et al., 2021; Vasavada et al., 2017; Wang et al., 2010; Zhu et al., 2023). The activation of those areas was significantly correlated with olfactory and cognitive functions (Wang et al., 2010). Reduced task-related engagement of the ON and decreased suppression of DMN activity have also been described in MCI and AD patients (Lu et al., 2019), highlighting a selective vulnerability of olfactory and resting-state networks along the AD continuum.

Previous studies have aimed to identify neuroimaging predictors of conversion from MCI to AD dementia, demonstrating complex and variable patterns of altered resting-state connectivity—particularly within the DMN (Eyler et al., 2019). Imaging predictors of MCI-to-AD conversion have been identified in the medial temporal lobe (Gullett et al., 2021), with more recent neuroimaging studies incorporating machine learning methods and demonstrating reliable predictive value using fMRI features (Valizadeh et al., 2025). Finally, evidence from a recent meta-analysis did not find strong associations between olfactory function and amyloid-*β*/tau burden (Tu et al., 2020), highlighting the complexity of olfactory dysfunction in AD, and suggesting that olfactory performance alone may not sufficiently explain functional connectivity changes.

Taken together, these findings demonstrate that both structural and functional alterations within olfactory-related regions emerge early in the AD continuum and may contribute to the variability observed in olfactory performance. However, despite increasing evidence, rs-fMRI alterations alone do not currently represent useful biomarkers of progression to dementia. Recent evidence suggests that combining resting state connectivity measures with olfaction testing may offer a more promising approach for identifying early indicators of clinical progression (Xie et al., 2024). On this rationale, the present study aimed to investigate whether resting-state functional connectivity of the Olfactory Cortex (OC)— considered as a proxy of ON integrity— could help distinguish, at baseline, between MCI who remained clinically stable (sMCI) or converter to AD dementia (cMCI) during a 4 (±1.6)-year follow-up. Given that rs-fMRI is easier to implement than task-based paradigms—especially in neurodegenerative disorders where understanding instructions may be influenced by the patient’s cognitive status—we focused on the OC as seed region to explore its functional connectivity in the early phase of disease progression. Finally, we assessed the relationship between olfactory performance and OC functional connectivity to determine whether specific connectivity patterns may reflect early olfactory-related neural vulnerability associated with conversion risk.

## 2 Materials and methods

### 2.1 Participants and timeline of the study

Thirty amnestic-MCI patients (16 males; mean age ± standard deviation, 70.4 ± 8) were recruited from the Cognitive Neurology Clinic of the Azienda Ospedaliero-Universitaria of Modena, Italy. The patients underwent neurological examination and neuropsychological assessment at the time of the MCI diagnosis (T0) and at follow-up (T1) of at least 2 years. The degree of cognitive impairment was assessed by the Mini-Mental State Examination (MMSE; Folstein et al., 1975). At T0, patients underwent also an olfactory function evaluation and a multimodal MRI protocol. MCI diagnosis was established according to the updated Petersen criteria (Petersen et al., 2014; Petersen, 2016). Participants were required to meet all of the following: (i) subjective cognitive concern, reported by the participant and/or an informant; (ii) objective cognitive impairment, defined as performance ≥1.0–1.5 SD below age- and education-adjusted norms in amnestic cognitive domain; (iii) preserved functional independence, operationalized as intact basic activities of daily living and no clinically significant impairment in instrumental activities of daily living (IADL scores within normal limits; Katz et al., 1963; Lawton and Brody, 1969); (iv) absence of dementia, based on clinical interview and global cognitive screening (MMSE and/or MoCA) not consistent with dementia-level impairment. AD diagnosis followed the NIA–AA criteria (McKhann et al., 2011). All patients included in the AD group met criteria for probable AD dementia, defined by: (i) insidious onset and progressive decline in cognition, with prominent episodic memory impairment documented by neuropsychological assessment; (ii) cognitive deficits in at least two domains, severe enough to interfere with usual daily functioning; (iii) exclusion of other causes of cognitive impairment (e.g., significant cerebrovascular disease, frontotemporal degeneration, major psychiatric conditions, or metabolic/systemic disorders) through clinical evaluation and routine laboratory tests. A prior, current, or past history of other neurological diseases, neurosurgery, or major psychiatric disorders were considered exclusion criteria. Participants were screened for acute or chronic nasal conditions (e.g., rhinitis, sinusitis) and for temporary upper respiratory infections; individuals presenting any such conditions at baseline and smokers were excluded to ensure that olfactory performance was not influenced by peripheral factors. Demographic and cognitive characteristics are reported in Table 1. The study was conducted according to the 2013 version of the Declaration of Helsinki and had been approved by the Ethics Committee of the Area Vasta Emilia Nord (protocol number: 107/2016/SPER/AOUMO), with all subjects providing their written informed consent before participating in the study.

**Table 1:** Demographic, cognitive characteristics, and olfactory functions scores of patients.

	Whole group (*n* = 30)	cMCI (*n* = 15)	sMCI (*n* = 15)	Group comparisons
Age (years)	70.4 (±8)	71.9 (± 7.3)	68.9 (± 9)	*p* = 0.3
School age	10.5 (± 4)	10.1 (± 4.1)	10.9 (± 3.9)	*p* = 0.65
Gender F:M	14:16	7:8	7:8	*p* = 1
MMSE baseline	27 (± 2)	26.6 (± 2.2)	27.5 (± 1.8)	*p* = 0.24
Sniffin’ Sticks—Smell test	(*n* = 29)	(*n* = 14)	(*n* = 15)	
Threshold test	6.7 (± 3)	7.3 (± 2.9)	6.2 (± 2.9)	*p* = 0.3
Discrimination test	8.5 (± 2.5)	7.6 (± 2.0)	9.4 (± 2.6)	*p* = 0.046
Identification test	9.1 (± 2.8)	8.5 (± 3.1)	9.7 (± 2.3)	*p* = 0.23
Total score	24 (± 5.6)	22.7 (± 5.9)	25.3 (± 5.2)	*p* = 0.2

Mean values and standard deviation values (in parenthesis) are reported. MMSE, Mini Mental State Examination.

### 2.2 Olfactory assessment

The Sniffin’ Sticks (Burghart®, Wedel, Germany) was administered at T0 to evaluate the patients’ olfactory function by means of three subtests: threshold, identification and discrimination. During threshold test, patients were presented with 16 triplets of pens. During the threshold assessment, participants were blindfolded in accordance with the standardized Sniffin’ Sticks protocol to prevent visual cues from influencing odor detection. One of the pens contained N-butanol or phenylethyl alcohol (BUT/PEA) diluted in a solvent according to decreasing concentrations, while two pens contained solvent only. Participants had to identify the BUT/PEA pen among the set of three. The identification test included 16 odors (Hummel et al., 1997). After 3–4 s of exposure to an odor, participants were asked to determine which of the four item cards best described the smell. The number of correct responses out of 16 represented the identification score. In the discrimination test, the participant was asked to identify which item had a different odor from the other two in each of 16 triplets of odors. The odor discrimination score is the number of correct responses out of 16 (Rumeau et al., 2016). The examiner presented each odor with a felt tip pen while using odorless gloves.

### 2.3 Follow-up and diagnostic classification

At follow-up, patients were categorized as sMCI if they remained clinically stable or as cMCI if they converted to AD. The clinical diagnosis of AD was made according to published criteria (McKhann et al., 2011) by neurologists expert in neurodegenerative and cognitive disorders (MT and AC). Functional status was assessed using both Activities of Daily Living (ADL) and Instrumental Activities of Daily Living (IADL) scales (Katz et al., 1963; Lawton and Brody, 1969) to further characterize participants’ everyday functioning.

### 2.4 Behavioral analyses

Data distribution was assessed using the Shapiro–Wilk test. According to their distributional characteristics, demographic data, MMSE score and Sniffin’ Sticks subtests, along with the total score (TDI), were compared between the sMCI and cMCI patient using T-test or chi-square (*χ*^2^) test. Group comparisons between Sniffin’ Sticks subtests scores were considered statistically significant if *p* < 0.0125, according to Bonferroni correction for *n* = 4, *α* = 0.05. The percentages of converter and stable patients with hyposmia, normosmia, and anosmia were calculated according to published normal values (Hummel et al., 2007; Rumeau et al., 2016).

The data were analyzed using JASP Software, version 0.18.3 (https://jasp-stats.org; Goss-Sampson, 2025).

### 2.5 fMRI protocol

MRI recordings were acquired using a 3 T Philips Achieva MR-scanner (Philips Healthcare, Best, The Netherlands). Resting state functional data consisted of a gradient-echo echo-planar sequence from 30 axial contiguous slices (TR = 2,000 ms, TE = 35 ms, in-plane matrix = 80 × 80, voxel size: 3 × 3 × 4, FOV = 240, total duration = 8 min, 240 volumes). Slices were acquired using an interleaved ascending slice acquisition order. Foam pads were used to improve the comfort of the subjects inside the coil and minimize possible head movements. All subjects were instructed to stay awake, and not to focus their thoughts on anything in particular, avoiding any structured mental activity (counting, rehearsing, etc.), and keeping their eyes closed. Several initial dummy scans were acquired, but they were automatically discarded by the scanner and not stored. A high-resolution T1-weighted anatomical image was also acquired for each participant to allow spatial normalization and anatomical localization. The volume consisted of 170 sagittal slices (TR = 9.9 ms, TE = 4.6 ms, in plane matrix = 256 × 256, voxel size = 1 mm isotropic).

### 2.6 fMRI data processing and analysis

MRI data were preprocessed and analyzed using MATLAB version R2020a (The MathWorks Inc., Natick, Mass) and SPM12 (Wellcome Department of Imaging Neuroscience, London, UK). Functional volumes of each participant were slice-timing corrected, realigned to the first functional volume acquired. The T1-weighted image was co-registered to the mean functional image and segmented using standard SPM’s tissue probability maps. The estimated deformation fields’ warp parameters (standard SPM segmentation) were used to normalize to the Montreal Neurologic Institute (MNI) template implemented in SPM12. A temporal filter (0.01–0.08 Hz) was applied to the voxel-wise BOLD time series, prior to nuisance regression, to reduce low frequency drifts and high frequency physiological noise. Finally, functional volumes were smoothed using a Gaussian kernel of FWHM = 6 × 6 × 8 mm^3^.

#### 2.6.1 Seed-based functional connectivity analyses: olfactory cortex

Functional connectivity maps were obtained using the voxel-wise approach by computing functional connectivity patterns between two regions of interest (ROI) and each voxel within the brain. The AAL2 Atlas (Tzourio-Mazoyer et al., 2002; Rolls et al., 2015) was used to define two seed regions corresponding to the left and right olfactory cortex (OC), which encompass the piriform cortex and adjacent olfactory areas. For each participant, the mean BOLD signal time course at T0 was extracted from each seed region using MarsBaR.^1^

Two distinct first-level regression analyses were performed for each participant, one for the left and one for the right OC. Single-subject voxel-wise general linear models (GLM) were performed, with the seed region’s time course entered as the regressor of interest. The six rigid-body head-motion parameters (translation and rotation) and the mean signal time courses from white matter and cerebrospinal fluid were included as nuisance regressors. Global signal regression (GSR) was not applied, and no scrubbing or exclusion of motion outlier volumes was performed. Single patient contrast images were generated by estimating the regression coefficient between the left and right OC time series and the whole brain.

Contrasts images of each patient were then entered in a second-level full factorial design (2 × 2), with group (cMCI and sMCI) and seed laterality (left and right olfactory cortex) as factors. Age and sex were included as covariates of no interest to account for individual variability. Statistical maps were thresholded using voxel-wise *p* < 0.001 and cluster-extent thresholding to achieve whole-brain family-wise correction at *α* < 0.05 (Woo et al., 2014).

The cMCI versus sMCI contrast was used to extract beta values from the resulting functional clusters. Beta estimates represent the strength of BOLD signal covariation between the seed region and each voxel, were plotted for detailed examination, and were used as a measure of functional connectivity in correlation analyses with the Sniffin’ Sticks subtests scores. Bonferroni correction (*n* = 3, *α* = 0.05) was applied to all correlations to account for multiple comparisons, and *p*-values were considered statistically significant if *p* < 0.0167.

## 3 Results

Thirty MCI patients were considered for fMRI analysis; smell test scores for one cMCI were not collected. Therefore, the group with both behavioral and fMRI data consisted of 29 patients.

### 3.1 Behavioral data

Based on established normative values (Hummel et al., 2007), 3 cMCI patients (21%), and 1 sMCI patient (7%) met criteria for functional anosmia (total score ≤ 16.5). One cMCI patient (7%) and 3 sMCI patients (20%) fell within the normosmic range (total score > 30.5). The majority of participants—10 cMCI patients (71%) and 11 sMCI patients (73%) showed hyposmia (scores between 16.5 and 30.5). Discrimination scores tended to be lower in the cMCI group (7.6 ± 2.0) compared with the sMCI group (9.4 ± 2.6), although this difference did not reach statistical significance [t(27) = 2.094, *p* = 0.046]. Threshold, identification, and total scores were comparable between the groups (see Table 1 for details).

### 3.2 fMRI data

#### 3.2.1 Functional connectivity of the olfactory cortex in cMCI and sMCI

**Figure 1:**
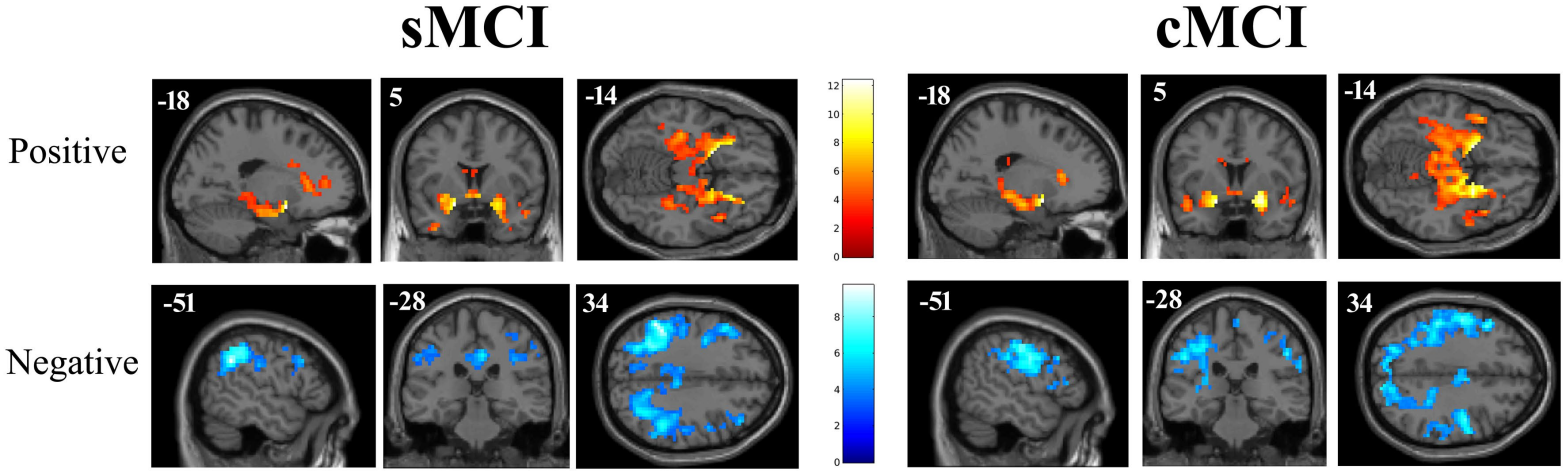
Positive (top) and negative (bottom) functional connectivity networks of the primary olfactory cortex in stable (left) and converter (right) MCI patients are shown in sagittal, coronal, and axial views (neurological convention) overlaid on the standard T1-weighted structural template implemented in SPM12. Results are displayed with a cluster size threshold *k* ≥ 31, corrected at *α* < 0.05. Color bars represent *t*-values.

Seed-based analyses revealed that the OC of both groups was functionally connected with the bilateral anterior insula, anterior cingulate cortex, middle frontal gyrus, hippocampus, amygdala, parahippocampal gyrus, precentral gyrus, and left lateralized clusters in the superior parietal lobule, as well as angular and supramarginal gyri (AG, SMG). The OC of sMCI group exhibited additional functional connectivity to the posterior cingulate cortex (PCC), bilateral inferior frontal gyrus (pars orbitalis), superior frontal gyrus, precuneus (PCU), supplementary motor area (SMA), and putamen, and caudate nucleus (Figure 1).

**Figure 2:**
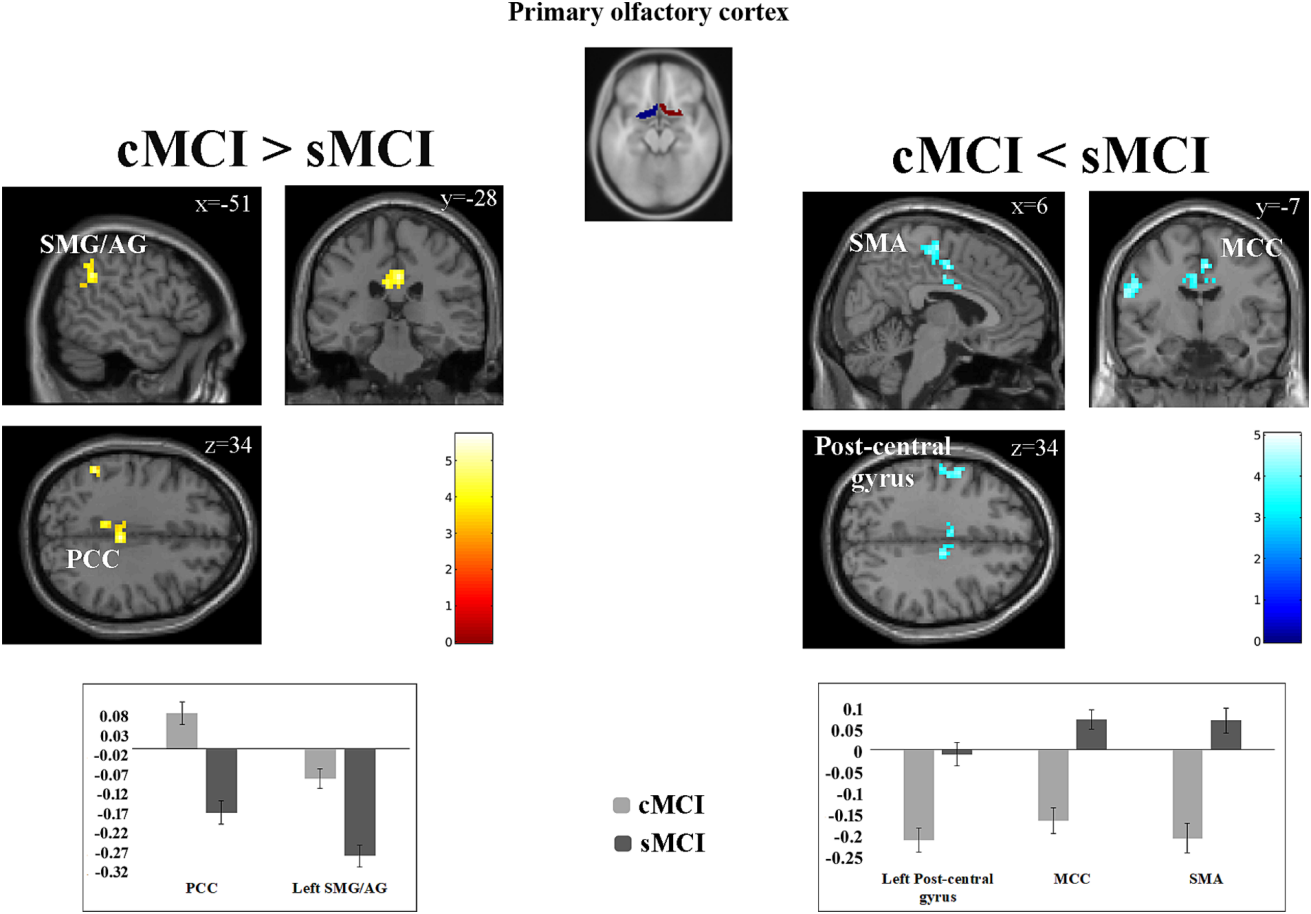
Functional connectivity differences between groups. Results of the cMCI > sMCI contrast (left) and cMCI < sMCI contrast (right), illustrate regions showing increased or reduced connectivity with the primary olfactory cortex in the cMCI group (cluster size threshold *k* ≥ 43; whole-brain corrected at *α* < 0.05). Significant clusters are overlaid on the standard T1-weighted anatomical template implemented in SPM12. Color bars represent *t*-values. Bar plots display the cluster-averaged GLM beta estimates for each group. SMG/AG, supramarginal and angular gyri; PCC, posterior cingulate cortex; SMA, supplementary motor area; MCC, mid-cingulate cortex.

Compared to sMCI, cMCI showed enhanced functional connectivity of the olfactory cortex with the PCC/PCU and reduced negative functional connectivity with the left SMG/AG. Decreased functional connectivity of the OC with the posterior mid-cingulate cortex (MCC), the SMA and left pre- and post-central gyri was also observed in cMCI compared to sMCI (Figure 2; Table 2).

**Table 2:** Areas of significant increased (cMCI > sMCI) and reduced (cMCI < sMCI) functional connectivity of patient’s olfactory cortex (cluster size threshold *k* ≥ 43, corrected at *α* < 0.05).

			Cluster	Voxel level	MNI coordinates		
Brain areas	BA	Side	K	T	*x*	*y*	*z*
cMCI > sMCI							
Cerebellum		L	48	5.72	−27	−64	−26
				4.83	−39	−64	−30
				4.75	−27	−73	−26
Posterior cingulate cortex	23	L/R	75	5.17	3	−28	34
				4.4	−6	−28	30
				4.19	−6	−40	38
Supramarginal and angular gyrus	39	L	43	4.79	−51	−49	34
				4.06	−33	−55	42
				3.8	−39	−49	42
cMCI < sMCI							
Mid-cingulate cortex (posterior division)	23	L/R	45	5.03	12	−13	38
				4.54	−6	−4	38
				4.06	0	−1	26
Precentral gyrus, supplementary motor area	4, 6	R	60	4.84	3	−25	58
				4.54	6	−7	46
				4.36	6	−16	62
Postcentral gyrus	1, 2, 3	L	81	4.7	−54	2	26
				4.42	−54	−13	42

BA, Brodmann area; L, left; R, right.

#### 3.2.2 Correlation analyses

Beta values of left SMG/AG were negatively correlated with discrimination score, regardless of the group (*r* = −0.59; *p* < 0.001). No significant correlations were identified between beta values extracted from PCC, SMA, MCC, postcentral gyrus and the threshold, discrimination and identification scores.

## 4 Discussion

The aim of the present study was to investigate whether the functional connectivity of the olfactory cortex is already altered at the time of diagnosis of MCI, and whether it predicts future conversion to AD dementia. We found that in MCI converter to dementia (in comparison to stable MCI), the functional connectivity of the OC with medial regions (posterior MCC, SMA) and left sensory-motor areas was decreased. Meanwhile, it was increased with PCC/PCU and less negative with the left inferior parietal lobule (SMG/AG). The increased functional connectivity between the OC and the SMG/AG was also negatively correlated with discrimination performance.

Olfactory dysfunction increases dementia risk, particularly in combination with AD genetic susceptibility (Laukka et al., 2023), but the neural underpinnings of this mechanism remain unclear. The OC, a key region of the Olfactory Network, is one of the olfactory-related regions which is more vulnerable to structural and functional modifications in MCI and AD dementia (Steffener et al., 2021; Vasavada et al., 2015, 2017; Wang et al., 2010). We found reduced functional connectivity of the OC with MCC, SMA and sensory-motor areas. The posterior part of the MCC plays a primary role in reflexive orientation of the body in space to sensory stimuli, a function that is critically modulated by parietal afferents projections (Vogt, 2016). It is functionally connected with sensorimotor networks (Yu et al., 2011). A significant difference in the functional connectivity pattern of MCC with motor and premotor regions has been reported in MCI patients compared to the healthy subjects (Cera et al., 2019). In the present study, the lower functional connectivity of MCC, SMA and post-central region with the OC suggest the involvement of these regions in olfactory dysfunction of MCI converter to AD dementia. We speculate that it may be due to the role of these regions in orienting to sensory, including olfactory stimuli.

Increased OC’s functional connectivity emerged with the PCC/PCU, critical components of posterior DMN, a network known to be vulnerable in the AD continuum (Eyler et al., 2019). Prior evidence indicates functional coupling between olfactory processing and DMN hubs, as demonstrated by reduced task-related functional suppression of DMN during olfactory fMRI in patients with AD dementia compared to healthy controls (Lu et al., 2019). The significance of the PCU in olfaction is further underlined by a study involving dementia-free individuals, both with and without olfactory identification deficits, which revealed functional connectivity changes of the PCU in subjects experiencing olfactory impairment (Xie et al., 2024). Therefore, the enhanced OC–PCC coupling observed in cMCI may reflect early disruption of large-scale networks integrating olfactory, attentional and mnestic processes.

Our data revealed that functional connectivity of the OC was increased also with the SMG and AG, along with a negative correlation of these regions with the patients’ performance at odor discrimination. The AG serves as cross-modal hub where multisensory information are combined and integrated (Seghier, 2013). Our data align with previous research showing significantly stronger dynamic functional connectivity from the OC to the AG in individuals with acquired anosmia compared to healthy controls, as well as a significant negative correlation between these functional connectivity values and the total score of the Sniffin’s Sticks (Iravani et al., 2021). The results of our study support the hypothesis of Iravani et al. (2021) that the functional connectivity changes between OC and AG, as well as with the PCC, acts as a sensory compensatory mechanism. This hypothesis is further supported by the negative correlation between the functional connectivity of the AG and olfactory discrimination performance that we identified in the present study, reinforcing the findings of Iravani et al. (2021).

Although sMCI and cMCI participants showed comparable olfactory performance at baseline, group differences in olfactory-cortex functional connectivity may index alterations that emerge very early in the disease course, potentially preceding robust or clinically overt olfactory deficits. Converging evidence indicates that the olfactory system is affected at prodromal stages of AD, with early neuropathological and network-level changes in olfactory regions that anticipate marked olfactory decline and dementia onset (e.g., Lu et al., 2019; Yan et al., 2022; Li et al., 2025). In this framework, disrupted olfactory-related FC in cMCI individuals may reflect prodromal circuit vulnerability that is only partially captured by conventional psychophysical testing. Moreover, these results emphasize the utility of FC alterations as highly sensitive neuroimaging indicators for detecting individuals at elevated risk during the prodromal stages of Alzheimer’s disease (Zhang et al., 2024; Zhu et al., 2025).

## 5 Limitations and future directions

Our work has the following limitations. First, the relatively small sample size and the short follow-up period for some participants may have reduced the statistical power of our analyses. Increasing both sample size and follow-up duration would allow a more robust investigation of the relationship between olfactory performance and rs-fMRI measures and strengthen the clinical significance of the findings. Larger and more balanced cohorts would also enable clearer comparisons between MCI individuals with olfactory impairment who convert to AD dementia and normosmic individuals who remain stable. Moreover, extending the analysis beyond olfactory cortical regions may provide a more comprehensive understanding of early olfactory-related network alterations in MCI and AD. Additionally, several clinical and demographic variables—such as depressive symptoms, comorbidities (e.g., hypertension, diabetes, vascular disease)—were not systematically collected, limiting the characterization of the sample. Future studies should include these measures to better account for potential modulators of olfactory function and their interaction with neurodegenerative processes.

## 6 Conclusion

In conclusion, our results support the association between resting-state data and olfaction testing as promising indicators for detecting early and preclinical signs of conversion from MCI to AD dementia. Our results (i) align with previous studies showing functional changes in olfactory-related regions in MCI and patients with AD dementia, and (ii) support a strong interplay between the olfactory network and DMN, along with an early selective vulnerability of ON and DMN in the AD continuum. Moreover, a critical role of the AG emerged from our results, and we speculate that the negative correlation between olfactory ability and the functional connectivity of this multisensory integration region may serve at the time of MCI diagnosis as a marker of progression to AD dementia.

## Data Availability

The raw data supporting the conclusions of this article will be made available by the authors, without undue reservation.

## Footnotes

1
http://marsbar.sourceforge.net
